# Loot

**Published:** 1871-03

**Authors:** 


### Loot.

### *Poisoning by Carbolic Acid.*

The *Canada Med. Journal* reports a case from the Montreal General Hospital, where a patient manifested symptoms of the toxical effects of carbolic acid by external application. He had been suffering from acute eczema of the entire cutaneous surface for some five weeks. All applications having proved ineffectual, an ointment of carbolic acid and lard, one part to four, was applied to the arms and thighs. An hour and a half after the application, the patient was found apparently moribund. Prompt cleansing of the surface, and internal administration of stimulants, both by mouth and rectum, restored him. Five hours later free vomiting occurred, and the vomited matters gave off a strong odor of carbolic acid. Upon recovery, the patient stated that a few minutes after the application of the ointment he realized "a peculiar burning, pricking sensation over the whole body," accompanied by extreme vesical tenesmus, and then followed a state of perfect insensibility.

### *Intermittent Fever successfully treated by th Iodide of Potassium after Quinia had failed.*

Dr. S. L. Abbott records (Boston Medical & Surgical Journal, Oct. 7, 1869), a case of this kind: A young man had had repeated attacks of intermittent fever. During his last attack "the chills had recurred daily, and the patient had suffered much from almost constant, deep-seated pains of a rheumatic character, mostly in the chest and arms, which were most severe in the latter part of the day and at night, sometimes seriously disturbing sleep. There was some tenderness on pressure over the spleen, but no enlargement of that organ could be felt. Appetite much impaired. ℞.—Potass. iodid., gr. v; Fl. ext. quassiæ, f℥ss before each meal.

"May 18. Patient reported that he commenced the use of the medicine on the 14th. On the evening of that day he had a severe chill, which lasted two hours, and was followed by fever and profuse sweating, as usual. On the 15th he had another attack, but much less severe. There had been no recurrence since. The appetite was improving; the bowels were regular, and the patient felt much better generally. Directed to continue the use of the medicine until the 21st, when the evening dose to be omitted.

"June 3. No chill since last report. Appetite said to be 'enormous,' 'better than for three years.' Patient says, 'the medicine killed the ague in just two days.' The pain in the bones ceased after the third day."

